# Brazilian Protocol for Sexually Transmitted Infections 2020: syphilis diagnostic tests

**DOI:** 10.1590/0037-8682-630-2020

**Published:** 2021-05-17

**Authors:** Pâmela Cristina Gaspar, Álisson Bigolin, José Boullosa Alonso, Esdras Daniel dos Santos Pereira, Maria Luiza Bazzo

**Affiliations:** 1 Ministério da Saúde do Brasil, Secretaria de Vigilância em Saúde, Departamento de Doenças de Condições Crônicas e Infecções Sexualmente Transmissíveis, Brasília, DF, Brasil.; 2 Universidade de Brasília, Programa de Pós-Graduação em Saúde Coletiva, Brasília, DF, Brasil.; 3 Universidade Federal de Santa Catarina, Laboratório de Biologia Molecular, Microbiologia e Sorologia, Florianópolis, SC, Brasil.

**Keywords:** Syphilis, Neurosyphilis, Congenital syphilis, Diagnosis

## Abstract

The recommendations for diagnostic tests for investigating syphilis are part of the Clinical Protocol and Therapeutic Guidelines for Comprehensive Care for People with Sexually Transmitted Infections and the Technical Manual for Syphilis Diagnosis, published by the Brazilian Ministry of Health. These recommendations were developed based on scientific evidence and discussions with a panel of experts. This article presents direct tests to detect *Treponema pallidum* in lesions and algorithms that combine treponemal and non-treponemal antibody tests to assist in syphilis diagnosis, with the aim of contributing to the efforts of health service managers and health professionals in qualifying health care. The article also covers the use of non-treponemal tests to investigate neurosyphilis and guidelines for interpreting non-treponemal antibody titers in monitoring the treatment and diagnosis of congenital syphilis, as well as prospects for innovations in diagnosis. The critical role of rapid immunochromatographic treponemal tests for public health and for addressing syphilis is also highlighted.

## Abstract (key points)

**Highlighted excerpt:** During the natural evolution of syphilis, activity periods with distinct clinical, immunological, and histopathological characteristics are interspersed with latent periods when there are no signs or symptoms, making access to tests critical for early diagnosis.

## FOREWORD

This article relates to the recommendations for diagnostic tests for investigating syphilis, which is part of the Clinical Protocol and Therapeutic Guidelines for Comprehensive Care (PCDT), for People with Sexually Transmitted Infections (IST)^1^ and the Technical Manual for Syphilis Diagnosis^2^. For the development of the PCDT, we selected and analyzed the available pieces of evidence in the literature and held a panel discussion with specialists to develop the recommendations. The manual was approved by ministerial ordinance^3^, and the PCDT was approved by the National Committee for Technology Incorporation into the Brazilian National Health System (Conitec)^4^ and updated by a group of specialists in STI in 2020^1^. 

## INTRODUCTION

Syphilis is a STI caused by *Treponema pallidum*, a human-exclusive bacterium whose transmission occurs through sexual contact and vertical transmission. It can rarely be transmitted through blood transfusion or occupational accident^1^ ^,^ ^5^ ^-^ ^8^. 

During the natural evolution of the disease, there are activity periods with distinct clinical, immunological, and histopathological characteristics interspersed with latent periods when there are no signs or symptoms. This fact makes constant access to tests critical for helping early diagnosis^1^. Congenital syphilis is one of the most significant challenges in prenatal care, posing a need for pursuing correct diagnosis and treatment during pregnancy for vertical transmission prevention^1^ ^,^ ^8^ ^,^ ^9^.

Despite the availability of treatment since the late 1930s and the lack of resistance of *T. pallidum* to penicillin, syphilis still represents a worldwide public health problem, with a growing epidemic trend, mainly in developing countries. In 2016, approximately 6.3 million new cases were reported worldwide^10^. In Brazil, in 2018, 158,051 cases of acquired syphilis and 62,599 cases of syphilis in pregnant women were reported. In the same year, 26,219 cases of congenital syphilis and 241 deaths occurred, with an incidence rate of 9/1,000 live births and a mortality rate of 8.2/100,000 live births^11^.

Regarding the Brazilian National Health System (SUS), the challenge of the universalization of access to healthcare action and services is comprised by technology incorporation, management of diagnosis and treatment inputs, and the standardization of clinical and laboratory guidelines and parameters. They materialize in critical components for comprehensive care, surveillance, control, and answer to syphilis for its historical condition^1^ ^,^ ^2^, the increasing number of cases, and its direct impact on maternal and child mortality^11^. This fight was established as an objective of signed international treaties and national commitments.

This article aims to systematize and update the contents of the national guidelines for the qualification of syphilis testing and diagnosis practices. 

## TYPES OF DIAGNOSTIC TESTS FOR SYPHILIS INVESTIGATION

Syphilis diagnosis is based on tests for direct pathogen detection or immunological tests^2^ ^,^ ^6^ ^-^ ^8^. Although the pathogen that causes syphilis is a bacterium, in vitro cultivation is still complex, and its use is not feasible in diagnosing the infection^2^.

Direct detection is useful for diagnosing primary and early congenital syphilis, and it helps in secondary syphilis diagnosis, as these steps of infection present lesions on the skin or the mucosa containing exudate with large amounts of the pathogen^1^ ^,^ ^2^. The methods for directly detecting *T. pallidum* include microscopy techniques and nucleic acid amplification test (NAAT), which presents the advantage of being positive from 1 to 3 weeks before the immunological tests^8^.

Dark-field microscopy aims to identify *T. pallidum* based on its characteristic morphology and motility in samples analyzed immediately after collection. Although it is a low-cost methodology, this analysis requires a microscope with a dark-field condenser and plate analysis experienced professionals, which can limit its use^12^ ^-^ ^14^. In addition, stained samples and direct immunofluorescence microscopy have rarely been performed in Brazil, as silver staining for detection of spirochaetes presents low sensitivity, unspecific for *T. pallidum* ^15^, and the inputs for fluorophore marking are each time scarcer^16^.

NAAT presents a good performance for detecting *T. pallidum* in samples of lesions, tissues, and liquor, and it can be an alternative for diagnosis. In Brazil, there are validated and registered methodologies for investigating *T. pallidum* in genital ulcers, and they are being analyzed for incorporation into the SUS^17^ ^,^ ^18^. 

Immunological tests detecting antibodies in whole blood, serum, or plasma samples are the most used for diagnosing syphilis, and they can be classified as treponemal and non-treponemal tests^1^ ^,^ ^2^. 

Non-treponemal tests detect anticardiolipin antibodies (IgM and IgG) through a flocculation reaction, in which they are linked to the micelles in the antigenic suspension composed of cardiolipin, lecithin, and cholesterol. Such tests are semiquantitative, and the reacting samples need to be diluted (factor 2), with result issuance as per the last titers with reactivity (e.g., 4, 8...128) or dilution (e.g., 1:4, 1:8...1:128). Non-treponemal tests do not have a cut-off point for defining syphilis. Consequently, any titer must be investigated for syphilis^1^ ^,^ ^2^ ^,^ ^14^. 

Among the non-treponemal tests, the first one to be standardized was the Venereal Disease Research Laboratory (VDRL) test, which uses the previously mentioned standard antigen preparation. The antigen was later changed with the addition of choline chloride and ethylenediaminetetraacetic acid, giving room to the unheated serum reagin (USR), which presents higher stability in antigen suspension and samples do not require heat inactivation. Another change in the antigen suspension was the incorporation of coal particles in the rapid plasma reagin (RPR) test, which allows for the amplification of flocculation, eliminating the need to read the results under a microscope. The toluidine red unheated serum test (TRUST) uses toluidine red particles instead of coal in the antigen suspension composition^2^ ^,^ ^6^ ^,^ ^8^ ^,^ ^14^.

All samples submitted for non-treponemal tests must be tested in pure and diluted forms to eliminate false non-reagent results due to the prozone phenomenon when there is an imbalance between the antigen and antibody quantity in the reaction^2^ ^,^ ^8^ ^,^ ^19^ ^,^ ^20^. False reagent reactions in non-treponemal tests for syphilis may also occur, as anticardiolipin antibodies may be produced due to other diseases that also cause cell destruction, such as systemic lupus erythematosus, chronic hepatitis, malaria, and Hansen's disease^8^ ^,^ ^19^ ^,^ ^20^.

Non-treponemal tests are useful for investigating active syphilis and treatment monitoring by comparing diagnosis titers with post-treatment titers. These tests present decreased positivity in primary syphilis, late latent syphilis, and tertiary syphilis, as they react approximately 6 weeks after the infection and tend to decrease the reactivity in the late stages of the disease, even without treatment^5^.

Treponemal tests are based on the detection of antibodies produced by the host in an immunological response (IgM and IgG antibodies) to the antigenic components of *T. pallidum*, such as fluorescent treponemal antibody absorption (FTA-Abs), *T. pallidum* particle agglutination (TPPA), *T. pallidum* hemagglutination assay (TPHA), enzymatic immune assays, and their modifications, in addition to rapid diagnostic tests^2^ ^,^ ^5^ ^,^ ^8^.

**FIGURE 1:**
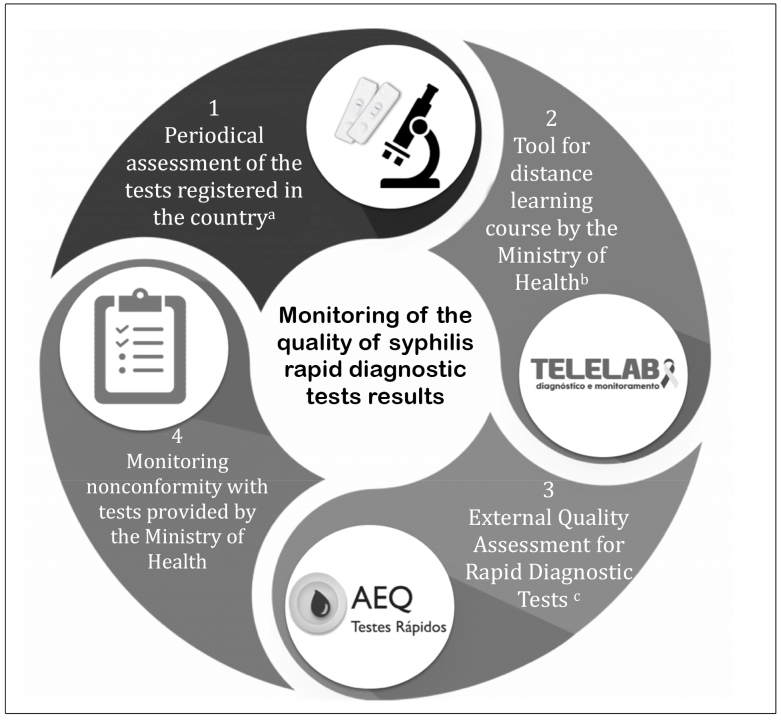
Monitoring of the quality of syphilis rapid diagnostic tests results by the Ministry of Health.

**Notes:** a) The tests assessed and the analysis methodology are published in scientific articles^43^; b) Information on the platform methodology and the courses is available on the website http://www.telelab.aids.gov.br; c) Information on the organization, scope, and criteria for assessment is available on the website http://www.qualitr.paginas.ufsc.br

Rapid diagnostic tests are easy to perform, do not require laboratory infrastructure, and can be performed by a skilled person. They are widely used in primary health care, maternity services, and places with problematic access to laboratories, and as they provide results within 30 min, they eliminate the risk of loss of user for non-return to care^1^ ^,^ ^2^ ^,^ ^8^. The better performance of rapid diagnostic tests is directly related to the training of professionals and rigorous compliance with all the steps set by the manufacturer, including kit storage, sample collection, test performance, and result interpretation. In addition, it is imperative to set quality assurance procedures in results obtained with syphilis rapid diagnostic tests^2^ ^,^ ^8^. In Brazil, the Ministry of Health monitors the quality of test results as shown in Figure 1, where there are (1) periodic assessment of the accuracy of the tests registered in the country, including rapid diagnostic tests, in partnership with reference laboratories; (2) Telelab platform availability, offering a distance learning course for healthcare professionals, with video classes and manuals presenting content on general information on infections, guidelines for the diagnosis and rapid diagnostic test procedure; (3) the External Quality Assessment for Rapid Diagnostic Tests program, which assesses the knowledge of professionals on the guidelines for the diagnosis and the quality of rapid diagnostic test performance, with educational and not punitive purposes; and (4) monthly monitoring of possible nonconformity with rapid diagnostic tests provided by the Ministry of Health in healthcare routine.

Treponemal tests are the first immunological tests to become positive, and they present better sensitivity and specificity than non-treponemal tests^5^ ^,^ ^8^ ^,^ ^21^. Treponemal tests cannot be used to differentiate active infection from a past one, and they are not useful for monitoring treatment, as most individuals with syphilis produce treponemal antibodies that persist throughout their lives, even after treatment^8^ ^,^ ^16^. Therefore, even after proper treatment, treponemal tests tend to be positive in most individuals. 

Treponemal tests detecting only specific IgM antibodies are not recommended for syphilis diagnosis, although they appear in the first post-infection humoral immune response, they are also found in latent periods and late stages, limiting the value of its detection in diagnosis, in addition to IgM detection presenting low sensitivity (50%)^5^ ^,^ ^8^.

## USE OF TESTS FOR SYPHILIS INVESTIGATION

To define syphilis diagnosis, we need to relate clinical data, results from diagnostic tests, past infection history, recent treatment records, and investigation of risk exposure^1^.

Syphilis diagnostic tests can be used to screening asymptomatic people or to investigate symptomatic patients. Test positivity can vary depending on the capacity for antibody production, the stage of infection, and the diagnostic test used^2^ ^,^ ^7^ ^,^ ^8^. 

In primary syphilis, when there is a typical ulcer (chancre) present, the visualization of treponemas can occur prior to seroconversion owing to the window period^5^ ^,^ ^6^ ^,^ ^8^. Immunological tests with negative results and persistence of infection suspicion must be repeated with a new sample after 30 days, for seroconversion assessment and monitoring of response to treatment, when so set^1^. 

In secondary syphilis, the positivity of immunological tests is 100% for practically all of them, and this period of infection has the highest titers in non-treponemal tests. In this phase, direct examinations may also be conducted (preferably molecular tests, when available) with samples from skin and mucosa lesions, which are very characteristic and rich in treponemas^5^ ^-^ ^8^. 

The evolution of the nontreated infection leads to a latent phase, in which the signs and symptoms disappear. In latent syphilis, treponemal tests remain highly positive, while in non-treponemal ones, the positivity starts to drop, leading to a decrease in the antibodies found and possible negative results^5^ ^-^ ^8^.

After the latent period, the infection may enter the tertiary stage, in which immunological tests behave in a similar way to latent syphilis. In this stage, treponemal investigation in various organs affected by treponema may also be helpful^5^ ^-^ ^8^.

## IMMUNOLOGICAL TEST ALGORITHMS FOR SYPHILIS DIAGNOSIS

**FIGURE 2:**
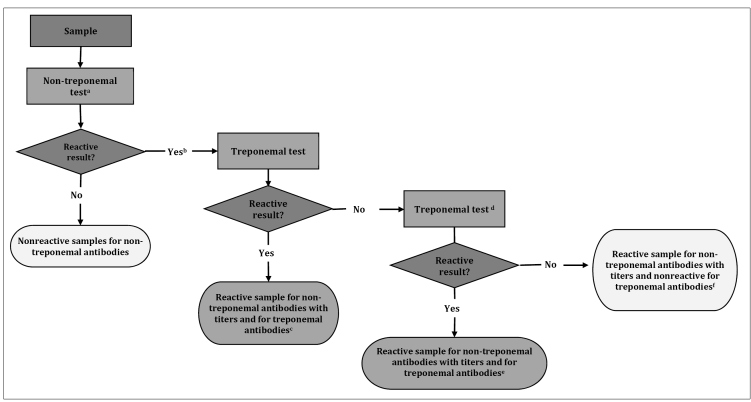
Algorithm with the classic approach for syphilis diagnosis (starting with non-treponemal test).

**Note:** a) Sample must be tested in pure and diluted form to eliminate the possibility of the prozone phenomenon; b) Sample must be diluted in factor 2 and undergo the non-treponemal test again. The results must be provided in titer values (e.g., 2, 4, 8...128) or the last dilution (e.g., 1:2, 1:4, 1:8...1:128) presenting reactivity; c) The detection of treponemal and non-treponemal antibodies suggests active syphilis; d) Treponemal test with a different methodology from the treponemal test already used in the second test algorithm. When the third test is not available, the results of each test must be released individually for assessment and clinical conduct; e) Detection of treponemal and non-treponemal antibodies suggests active syphilis. Probable false non-reagent results in the first treponemal test; f) Probable false-reaction result for syphilis in the non-treponemal test. Assess other clinical conditions besides syphilis that can generate reaction results in non-treponemal tests.

**FIGURE 3:**
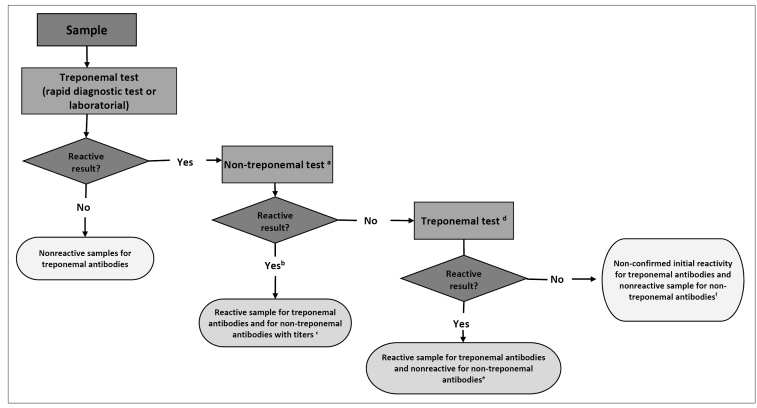
Algorithm with the reverse approach for syphilis diagnosis (starting with treponemal test).

**Note:** a) Sample must be tested in pure and diluted form to eliminate the possibility of the prozone phenomenon; b) Sample must be diluted in factor 2 and undergo the non-treponemal test again. The results must be provided in titer values (e.g., 2, 4, 8...128) or the last dilution (e.g., 1:2, 1:4, 1:8...1:128) presenting reactivity; c) The detection of treponemal and non-treponemal antibodies suggests active syphilis; d) Treponemal test with a different methodology from the treponemal test already used in the algorithm as the first test. If the third test is not available, the results of each test must be released individually for assessment and clinical conduct;e) Detection of treponemal antibodies only suggests recent syphilis or serological scarring. Assess exposure to risk, signs, symptoms, and history of syphilis treatment for defining clinical conduct; f) Lack of detection of non-treponemal antibodies and non-confirmation of treponemal antibody reactivity suggests no syphilis. Probable false reagent results in the first treponemal test.

The algorithms start with non-treponemal tests (classic approach, Figure 2) or treponemal tests (reverse approach, Figure 3) and may be automated, manual, or rapid. When the initial test performed is positive, it is necessary to conduct a second test. It must be a treponemal test in the classical approach or a non-treponemal test in the reverse approach. In situations where there is a discrepancy between the results of both tests, for better clinical guidance, the sample must undergo a third test with a different treponemal methodology from the previously conducted test. The report must contain the results of each test, with proper observation for the clinical professional, including the reactivity of non-treponemal tests in titers or dilution. This information is crucial for monitoring treatment and possible reinfection assessment^2^ ^,^ ^5^ ^-^ ^7^ ^,^ ^22^.

There is a preference for the algorithm with a reverse approach for investigating new cases, as treponemal tests become positive before non-treponemal tests^5^ ^,^ ^8^. However, in cases of syphilis history, we recommend starting an investigation with the classic approach, owing to the permanence of positivity in treponemal tests throughout life, in most cases of syphilis, regardless of whether they have been treated or not^2^ ^,^ ^7^.

When we conduct laboratory tests, the sample collection and delivery of results to the patient needs to happen as a priority in the assistance service, avoiding displacement of the patient to the laboratory. The setting of this flow and the use of rapid diagnostic tests, such as the first test, broadens access and adherence to healthcare^1^.

## TESTS FOR MONITORING SYPHILIS TREATMENT

Non-treponemal tests (e.g., RPR and VDRL) are used for monitoring syphilis after the treatment (cure control), as they tend to reduce their reactivity when the treatment is successful and, in cases of failure or reinfection, to increase the titers of the tests^5^ ^,^ ^6^ ^,^ ^13^. Good test practice recommends that the preferred method used in monitoring should be the same as that used for diagnosis and that the same laboratory performs them^2^ ^,^ ^6^. 

Testing with non-treponemal tests must be conducted at the beginning of the treatment (ideally on the first day), as the titers may significantly increase if the treatment is started only a few days after the diagnosis. The record of titers is useful as a base for clinical and laboratory monitoring^23^.

Only variations in the titers of non-treponemal tests of more or less than two dilutions are clinically relevant. Variations in result in only one dilution (e.g., RPR with 1:8 reactivity at diagnosis and 1:4 or 1:16 reactivity in treatment monitoring) may represent only a difference in laboratory interpretation^24^.

A non-treponemal negative test (seroreversion) may occur when the treatment is conducted in the earlier stages of the infection (primary syphilis and at the beginning of secondary syphilis). The decrease in titers in response to treatment may be slower in late syphilis treatment^25^.

When recent risk exposure is discarded, the persistence of non-treponemal test positivity results after adequate treatment, with a previous drop in titers in at least two dilutions is called a "serological scar" and does not characterize therapy failure. It is important to observe such criteria because low titers do not necessarily reflect a serological scar^5^ ^,^ ^7^ ^,^ ^8^.

Assessment of the presence of new signs and clinical symptoms, epidemiology (reexposure), and treatment history (duration, assessment, and therapy scheme) are fundamental, as it is difficult to differentiate between reinfection, reactivation, and serological scarring^1^.

## DIAGNOSTIC TESTS FOR NEUROSYPHILIS INVESTIGATION

*T. pallidum* bacteria spread to the central nervous system a few days after exposure^26^. Neurosyphilis may occur at any moment throughout syphilis, and it must not be considered only a manifestation of "tertiary" syphilis. The initial forms of neurosyphilis occur within months up to the first years after the primary infection, and they affect the meninges and blood vessels, while the late forms occur from years to decades after the primary infection, and they also affect the brain parenchyma and spinal cord^26^ ^,^ ^27^. 

Neurosyphilis diagnosis is based on a combination of clinical findings, alterations in the cerebrospinal fluid (CSF), and VDRL results in CSF. As there is no reference test (gold standard) with good sensitivity and specificity, neurosyphilis diagnosis continues to be a challenge in clinical practice^1^ ^,^ ^5^ ^,^ ^6^ ^,^ ^27^ ^,^ ^28^.

VDRL is the test chosen for investigating neurosyphilis^27^ ^-^ ^29^. VDRL sensitivity in CSF varies from 50% to 70%. Such values may be 30% lower when the test used is RPR^26^ ^,^ ^28^ ^,^ ^30^. A reacting VDRL in CSF permits a neurosyphilis diagnosis, although there is a possibility of finding false reagent results in some situations (e.g., trypanosomiasis, cerebral malaria, and meningeal carcinomatosis)^8^ ^,^ ^31^ ^-^ ^33^.

Even though they present a high sensitivity, treponemal tests are not very useful, as they keep reacting throughout life and present very variable specificity in CSF^28^. Therefore, we do not recommend the routine request of this test, especially in the current Brazilian epidemiological scenario^1^ ^,^ ^2^. 

It is difficult to find patients with neurosyphilis that do not present pleocytosis in CSF analysis, with the increase in lymphomonocytosis being the most common one^34^. However, CSF protein levels are not sensitive and specific for neurosyphilis, but its standardization is essential for post-treatment monitoring^34^ ^-^ ^37^. 

## DIAGNOSTIC TESTS FOR CONGENITAL SYPHILIS INVESTIGATION

For congenital syphilis diagnosis, we must assess the clinical-epidemiological history of the mother, conduct a detailed physical examination of the child, and assess the results of the laboratory tests and radiological examinations^1^ ^,^ ^38^.

When there is a history of syphilis in pregnant women, regardless of the treatment for the mother, we must compare the results of non-treponemal tests in the peripheral blood of the newborn and the mother, collected simultaneously, using the same method^1^. Non-treponemal tests in newborns cannot be conducted with a sample of the umbilical cord blood, given the mixing of newborn and maternal blood^1^ ^,^ ^2^ ^,^ ^6^. There is an indication of congenital syphilis only when the result of a non-treponemal test of the samples of the newborn is higher than that of the mother in at least two dilutions (e.g., mother 1:4, newborn ≥ 1:16)^1^ ^,^ ^2^ ^,^ ^5^ ^,^ ^8^.

When observing signs and symptoms in the child, we must also conduct direct examinations to investigate *T. pallidum* in samples of material collected from skin mucosal lesions or nasal secretion or, even, samples of biopsy or necropsy, when this is the case^5^ ^,^ ^8^.Children with congenital syphilis must be assessed with a series of additional examinations, highlighting CSF analysis, considering the previously described neurosyphilis diagnosis findings^1^. 

Treponemal tests must not be used up to 18 months of age, as before, there is no correlation between the positivity of treponemal tests in the newborn and the mother, which can suggest congenital syphilis^1^ ^,^ ^8^ ^,^ ^21^.Tests detecting IgM treponemal antibodies (e.g., FTA-Abs IgM and IgM enzymatic immunoassays) are not recommended for congenital syphilis diagnosis because although they do not penetrate the placental barrier, such antibodies are not detected in every case of congenital syphilis, which may imply nontreatment of children with syphilis^1^ ^,^ ^5^ ^,^ ^6^.

## FUTURE PERSPECTIVES ON INNOVATION IN SYPHILIS TESTING

We have achieved significant progress in diagnostic tests for various infections, including syphilis. Such progress has allowed for new approaches in diagnosing and monitoring the disease, increasing test access, and providing tools for helping to make decisions regarding patients.

Rapid diagnostic tests for the concomitant investigation of HIV and syphilis present good sensitivity for detecting antibodies against HIV, but they show moderate sensitivity (although it is proper) for detecting treponemal antibodies^39^. The use of such tests may optimize the routine of healthcare services and ensure syphilis tests in prenatal care as per the guidelines applied in Brazil, especially in areas with difficulties in accessing an efficient laboratory network^9^. 

Tests that can simultaneously detect non-treponemal and treponemal antibodies are already available for use. Systematic reviews suggest that the laboratory version of such tests is more sensitive and presents a specificity similar to that in traditional tests^40^. In contrast, rapid diagnostic non-treponemal and treponemal tests present reduced sensitivity compared to the laboratory methodologies for detecting non-treponemal antibodies^41^. They do not provide antibody titer values, making it difficult to differentiate between active syphilis and the process of cure^1^.

Automated RPR versions have already been developed worldwide, and validations of this methodology still need to be incorporated into the laboratory routine^42^.

Using multiplex NAAT allows for the simultaneous detection of one or more pathogens in a single sample, with little or no additional cost to the final value. It favors the timely diagnosis of other pathogens besides *T. pallidum*, with correct guidance on genital ulcer treatment^5^. 

## CONCLUDING REMARKS

The diagnosis of syphilis requires the correlation of clinical data, results from diagnostic tests, past infection history, recent treatment records, and investigation of risk exposure^1^. Diagnostic tests include direct examinations and immunological tests (treponemal and non-treponemal). Direct examinations are useful for the identification of *T. pallidum* lesions. Immunological tests must be performed as per conventional or reverse algorithms, combining two or more tests. Non-treponemal tests are also useful for monitoring the treatment and diagnosis of neurosyphilis and congenital syphilis. Decentralizing rapid diagnostic tests for primary health care services and maternity services may provide early diagnosis and adequate treatment for pregnant women and populations with increased vulnerability to syphilis.
